# Navigating migraine chronicity: the role of environmental risk factors and triggers

**DOI:** 10.3389/fneur.2025.1688987

**Published:** 2025-12-04

**Authors:** Hasan Doğan, Bilge Piri Cinar, Osman Iyilikci, Ezgi Uluduz, Sevim Eyüpoglu, Mustafa Iskender, Ummugulsum Kadioglu, Derya Uluduz, Aynur Özge

**Affiliations:** 1 Department of Neurology, School of Medicine, Samsun University, Samsun, Türkiye; 2 Department of Psychology, Manisa Celal Bayar University, Manisa, Türkiye; 3 School of Medicine, Koc University, Istanbul, Türkiye; 4 Department of Psychology, Brain 360 Holistic Approach Center, Istanbul, Türkiye; 5 Department of Neurology, Private Health Center, Kocaeli, Türkiye; 6 Department of Neurology, Istanbul School of Medicine, Istanbul University, Istanbul, Türkiye; 7 Department of Neurology, School of Medicine, Mersin University, Mersin, Türkiye; 8 NOROM Neuroscience and Excellence Center, Ankara, Türkiye

**Keywords:** chronic migraine, environmental factors, episodic migraine, treatment, migraine

## Abstract

**Purpose:**

Migraine is a prevalent and debilitating neurological condition that can significantly impact an individual’s quality of life. While various genetic and environmental factors contribute to the development and chronification of migraine, the role of specific environmental factors in the chronification of this condition remains inadequately explored. This article aims to address this research gap by focusing on the impact of manageable and unmanageable factors on the chronicity of migraine.

**Patients and methods:**

This cross-sectional observational study included 498 patients diagnosed with episodic or chronic migraine. Demographic characteristics of the patients were recorded on the demographic data form. Then, the patient’s headache characteristics (duration, type, presence of aura, accompanying features, etc.) and the answers to the questions about possible triggers of migraine and environmental factors were recorded in the data form.

**Results:**

In the chronic migraine group, oversleeping and stress were reported as triggers at a higher rate than in the episodic migraine group. In the episodic migraine group, it was found that participants who had TV transmitters were significantly less likely to benefit from treatment, while in the chronic migraine group, it was found that participants who had pets were significantly less likely to benefit from treatment.

**Conclusion:**

This study sheds light on the intricate relationship between environmental factors, migraine phenotype, and treatment response. By using comprehensive statistical methods and analyzing a rich dataset, we have gained valuable insights into the complexities of migraine management.

## Introduction

Migraines are among the top reasons for consulting a neurologist (1). Episodic migraine and chronic migraine can periodically turn into each other depending on many factors. Chronic migraine develops in 2.5% of patients with episodic migraine each year (2). Individuals with migraine can often identify the factors that trigger migraine attacks, but when these factors are not well defined, failure of medical treatments may result due to failure to manage risk factors.

The most common triggers of migraine attacks are defined as stress, auditory stimuli, fatigue, hunger, and menstrual periods (3). Another common point among triggers is changes in daily activity or environmental factors that increase migraine susceptibility (4). Kelman et al. examined triggers in 1,750 patients with migraine and determined that the most common triggers were stress, menstrual periods, long hunger periods, weather changes, sleep disorders, smell, alcohol, heat, and food (5). When multiple factors come together, they can increase the frequency of attacks in patients with rare migraines while also putting them at higher risk of triggering a migraine attack. Obtaining such trigger and headache information from large numbers of migraine patients may provide a better understanding of which triggers are common and/or strong and which may become important only when they act together (6). Studies focusing on this issue can provide data that may even enable the identification of individuals with migraine who share a set of common triggers.

Migraine attacks often begin as episodic events but may become chronic in some individuals over time. This can result from factors such as drug overuse, environmental influences, and failure to take necessary steps in lifestyle management. Exactly when chronicity begins and how it progresses in each individual cannot be predicted, as these outcomes are influenced by endogenous and exogenous factors as well as the individual’s genetic structure of the individual, and how it will progress cannot be predicted.

Studying the effect of triggers on migraine attacks is challenging, as potential triggers may interact, making statistical modeling difficult (7, 8). In a study evaluating episodic and chronic migraine groups along with trigger and environmental factors in 300 women with migraine, some triggers were identified, but no differences were observed between episodic and chronic migraine groups (9). However, a 2023 study specifically assessed endogenous and exogenous factors that may influence the chronicity of migraine (2).

In this study, the effects of triggers and environmental factors, which we identified with a comprehensive inquiry form in our migraine cases followed up in the same clinic, including episodic and chronic migraine cases, on both the pain phenotype and treatment response were evaluated.

## Materials and methods

### Participants

Patients over 18 years of age, who were followed up with a diagnosis of migraine and who had the cognitive level to provide information about demographic and clinical characteristics related to headache, were included. ICHD-3 criteria were used for the diagnosis of chronic migraine in the study group (1). Patients with headache more than 15 days per month for >3 months and at least 8 headaches with migraine characteristics were defined as “chronic migraine,” while those with headache less than 14 days per month were defined as “episodic migraine.” All treatment options for migraine and responses were recorded in detail.

### Study design

Ethical approval was obtained for this cross-sectional-observational study (2023/466), and the study was registered in clinical trials (NCT06304675). The International Headache Society (IHS) is involved in the development and publication of numerous guidelines for controlled treatment trials for primary headache disorders (7, 8). In the planning and implementation stages of this study, the principles of the clinical trials guideline of the International Headache Society (IHS) were followed, and the STROBE checklist was followed. Demographic characteristics of the patients were recorded on the demographic data form. Then, the patient’s headache characteristics (duration, type, presence of aura, accompanying features, etc.) and the answers to the questions about possible triggers of migraine and environmental factors were recorded in the data form.

### Statistical analysis

All analyses were performed with SPSS 28.0 software (IBM Corp., Armonk, NY, USA). The normality of data distribution was checked using the Shapiro–Wilk test and by investigating histograms and plots. Since data showed the normal distribution, values were presented as mean ± standard deviation (SD). An independent group’s *t*-test was used to examine whether various continuous measurements (e.g., attack frequency, attack duration, and attack severity) varied depending on the migraine groups and the presence or absence of environmental factors and triggers. However, in cases where the number of participants between groups is highly unbalanced, the Mann–Whitney U-test was used. Whether the presence or absence of environmental factors and triggers differed according to migraine groups was examined with chi-squared analysis. Similarly, whether the presence or absence of environmental factors and triggers changed in terms of response to treatment was also examined with chi-squared analyses. The analysis of environmental factors/triggers that tend to coexist according to migraine groups was performed by using hierarchical cluster analysis. As a result of the *post hoc* power analysis, a statistical power of 0.99 was calculated for the low effect size (0.2), 498 participants (264 in the episodic migraine group, 234 in the chronic migraine group), and the between-subject design.

Environmental factors and triggers obtained from all participants in the study were evaluated in detail, and two separate cluster analyses were conducted. To determine which factors that participants in the episodic and chronic migraine groups cited as triggers tended to coexist, a hierarchical cluster analysis was performed for both groups. Euclidean distance was used to calculate the distance between data points, and the full linkage clustering method was used to calculate the closeness of the clusters.

## Results

Four hundred and ninety-eight patients diagnosed with episodic and chronic migraine were included in the study. No significant difference was observed between the episodic migraine and chronic migraine groups in terms of age and gender. Participants in the chronic migraine group (10.3%) reported more allodynia than participants in the episodic migraine group (3.8%) (*p* = 0.004). The presence of any other accompanying symptoms does not differ according to migraine groups (all *p*s > 0.05). Demographic and clinical characteristics of the study group are detailed in Table 1.

**Table 1:** Demographic and phenotypic features of headaches in the study group.

	Episodic migraine *n* = 264 (52.8%)		Chronic migraine *n* = 234 (46.8%)		*p* ^a^	Total *n* = 498 (100%)	
	Female	Male	Female	Male		Female	Male
Age; mean ± *SD*	42.48 ± 10.23	41.84 ± 10.81	45.75 ± 11.54	45.84 ± 11.34	0.001	44.04 ± 10.98	43.61 ± 11.10
Education level; *N* (%)							
None	-----	-----	1 (0.5%)	-----	0.001^**b**^	1 (0.2%)	-----
Primary school	16 (7.4%)	3 (6.5%)	41 (20.3%)	6 (18.8%)		57 (13.6%)	9 (11.4%)
Secondary school	-----	-----	-----	1 (3.1%)		-----	1 (1.3%)
High school	54 (24.9%)	5 (10.9%)	33 (16.3%)	9 (28.1%)		87 (20.7%)	14 (17.7)
Graduate	99 (45.6%)	26 (56.5%)	89 (44.1%)	13 (40.6%)		188 (44.8%)	40 (50.6%)
Master’s degree	32 (14.7%)	8 (17.9%)	19 (9.4%)	3 (9.4)		51 (12.1)	11 (13.4)
PhD	1 (0.5%)	-----	-----	-----		1 (0.2%)	-----
Onset of headache disease (years); mean ± *SD*	22.65 ± 9.63	20.02 ± 9.70	22.77 ± 10.26	20.56 ± 8.79	0.728	22.68 ± 9.92	20.29 ± 9.23
MAD (severe pain) frequency; mean ± *SD*	6.29 ± 3.63	7.61 ± 3.81	15.20 ± 8.63	15.97 ± 8.49	0.000	10.66 ± 7.93	11.18 ± 7.51
HAD (mild pain) frequency; mean ± *SD*	1.25 ± 2.72	0.78 ± 2.17	7.5 ± 8.82	5.03 ± 8.41	0.000	4.27 ± 7.14	2.79 ± 6.46
Severity (VAS); mean ± *SD*	7.98 ± 0.93	8.07 ± 0.89	8.17 ± 0.92	8.28 ± 1.08	0.025	8.08 ± 0.93	8.17 0.97
Duration of attacks (hours); mean ± *SD*	11.09 ± 15.84	7.64 ± 6.97	12.63 ± 12.37	11.31 ± 10.47	0.113	11.86 ± 14. 27	9.10 ± 8.69
Unilateral Location; *n* (%)	115 (53%)	19 (41.3%)	98 (48.5%)	19 (59.4%)	0.800	213 (50.7%)	38 (48.1%)
Throbbing quality; *n* (%)	209 (96.3%)	39 (84.8%)	196 (97.0%)	29 (90.6%)	0.434	406 (96.7%)	68 (86.1%)
Associated symptoms *n* (%)							
Nausea	184 (84.8%)	40 (87.0%)	180 (89.1%)	28 (87.5%)	0.268	365 (86.9%)	69 (87.3)
Vomiting	59 (27.2%)	16 (34.8)	43 (21.3%)	8 (25.0%)	0.086	102 (24.3%)	24 (30.4%)
Photophobia	195 (89.9%)	39 (84.8%)	184 (91.1)	28 (87.5%)	0.645	380 (90.5%)	67 (84.8)
Phonophobia	175 (80.6)	34 (73.9%)	166 (82.2)	25 (78.1)	0.617	342 (81.4%)	59 (74.7%)
Osmophobia	89 (41.0%)	6 (13%)	79 (39.1%)	9 (28.1%)	0.799	168 (40%)	15 (19.0%)
Allodynia	7 (3.2%)	3 (6.5%)	24 (11.9%)	-----	0.004	31 (7.4%)	3 (3.8%)

^a^Comparison of episodic and chronic migraine groups.

^b^“None” and “secondary school” categories are not included in the analysis.

### Attack frequency environmental factors/triggers

When the monthly migraine day (MMD) frequency and the monthly headache day (MHD) frequency were compared according to the migraine groups, the MMD frequency and MHD frequency of the participants in the chronic migraine group were statistically significantly higher than the participants in the episodic migraine group (*p* < 0.001).

When the frequency of MMD was evaluated according to environmental factors, it was determined that the frequency of MMD did not differ significantly depending on the presence or absence of any environmental factor (all *p*s > 0.05). It was determined that the frequency of MMD differed significantly between the groups of female participants who specified menstruation among the triggers and those who did not [t(384) = 2.29, *p* = 0.023]. Additionally, the mean frequency of severe MMD in the group that stated smoking among the triggers (*M* = 12.61, *SD* = 8.63) was statistically significantly higher than the participants in the group who did not mention smoking among the triggers (*Z* = 2.26, *p* = 0.024) (Table 2).

**Table 2:** Comparison of MMD frequency according to the presence of triggers.

Triggers	*X̅* (*SD*)	*t*	*P*
Mensturation		2.29	0.023
Yes	9.92 (7.53) 12.06 (8.27)		
No			
Smoking		2.26	0.024
Yes	12.61 (8.63) 10.19 (7.50)		
No			

It was determined that the mean MHD frequency of the participants who stated that the TV transmitter was among the environmental factors was higher (*M* = 5.02, *SD* = 7.57) than the participants who stated that it was not present (*M* = 3.59, *SD* = 6.68, *Z* = 2.03, *p* = 0.042).

The frequency of MHD differed significantly between the groups that indicated physical activity, tea and coffee withdrawal, and stress, and those that did not indicate triggers (*p* = 0.006, 0.026, 0.010).

In line with the data obtained in this study, the frequency of MHD was significantly different in participants who reported at least three of the triggers (physical activity, hunger, stress, and insomnia) (*p* = 0.002).

It was determined that the frequency of MHD differed significantly between participant groups who did and did not indicate physical activity and travel together as triggers; *p* = 0.007. MHD frequency does not differ significantly depending on the presence or absence of other triggers (all *p*s > 0.05) (Table 3).

**Table 3:** Comparison of MHD frequency according to the presence of risk factors.

Triggers	*X̅* (*SD*)	*t*	*p*
TV transmitter		2.03	0.042
Yes	5.02 (7.57) 3.59 (6.68)		
No			
Physical activity		2.75	0.006
Yes	5.37 (8.21) 3.35 (6.42)		
No			
Stress		2.56	0.010
Yes	4.33 (7.36) 1.64 (4.52)		
No			
Tea and coffee withdrawal		2.75	0.006
Yes	5.47 (7.87) 3.60 (6.87)	2.25	0.026
No			
Physical activity, hunger, stress, and insomnia (three of them)		3.06	0.002
Yes	4.63 (7.49) 2.58 (5.95)		
No			
Physical activity and travel		2.74	0.007
Yes	5.65 (8.27) 3.44 (6.61)		
No			

### Attack duration environmental factors/triggers

When the attack durations of migraine patients included in the study were examined, it was determined that the average attack duration did not differ significantly between migraine groups and that the attack duration did not differ significantly depending on the presence or absence of any environmental factor (all *p*-values > 0.05).

### Evaluation of environmental factors/triggers in migraine groups

When migraine groups were compared according to the presence of environmental factors, the presence of a 200 m high voltage line (*p* = 0.041) and being in crowded places (*p* = 0.021) were found to be higher in the episodic migraine group. The participants in the chronic migraine group reported oversleeping and stress as triggers at a higher rate (respectively, *p* = 0.003, *p* = 0.032). The presence of any other triggers does not differ according to migraine groups (all *p*-values > 0.05). Other significant parameters are provided in Table 4.

**Table 4:** Comparison of migraine groups according to the presence of environmental factors/triggers.

Migraine group	200mt high voltage			
	Yes	No	*χ* ^2^	*p*
Chronic	27 (12.6%)	188 (87.4%)	4.19	0.041
Episodic	47 (19.7%)	192 (80.3%)		

	Oversleeping			
	Yes	No		
Chronic	133 (61.9%)	82 (38.1%)	8.95	0.003
Episodic	120 (48.0%)	130 (52.0%)		

	Stress			
	Yes	No		
Chronic	201 (93.5%)	14 (6.5%)	4.58	0.032
Episodic	219 (87.6%)	31 (12.4%)		

	Physical activity			
	Yes	No	*χ* ^2^	*p*
Chronic	27 (12.6%)	188 (87.4%)	4.19	0.041
Episodic	47 (19.7%)	192 (80.3%)		

	Crowded places			
	Yes	No		
Chronic	29 (13.1%)	192 (86.9%)	5.30	0.021
Episodic	51 (21.3%)	189 (78.8%)		

### Relationship between environmental factors/triggers and response to treatment

**Figure 1:**
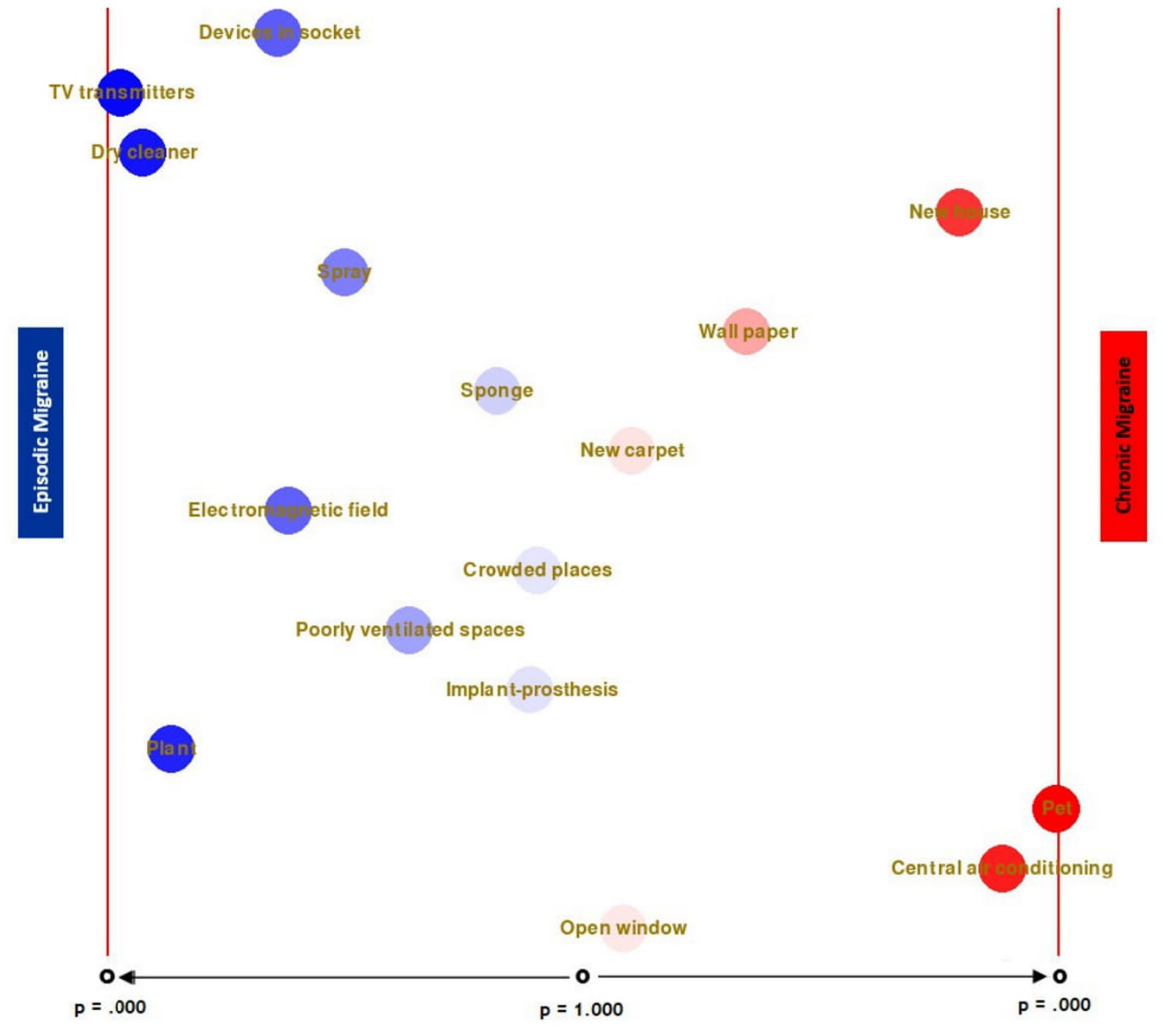
Comparison of environmental factors between the migraine groups and treatment response.

In the episodic migraine group, participants with TV transmitters were significantly less likely to benefit from treatment *χ*^2^ (1, *N* = 242) = 4.58, *p* = 0.032. On the other hand, in the chronic migraine group, it was found that participants who had pets were significantly less likely to benefit from treatment, *χ*^2^ (1, *N* = 218) = 7.20, *p* = 0.007. Apart from these, it was determined that the response to treatment did not change significantly depending on the presence of any other environmental factor. Figure 1 shows that, in the migraine group, the rate of not benefiting from treatment is higher in the presence of a certain factor, sorted according to their significance level.

**Figure 2:**
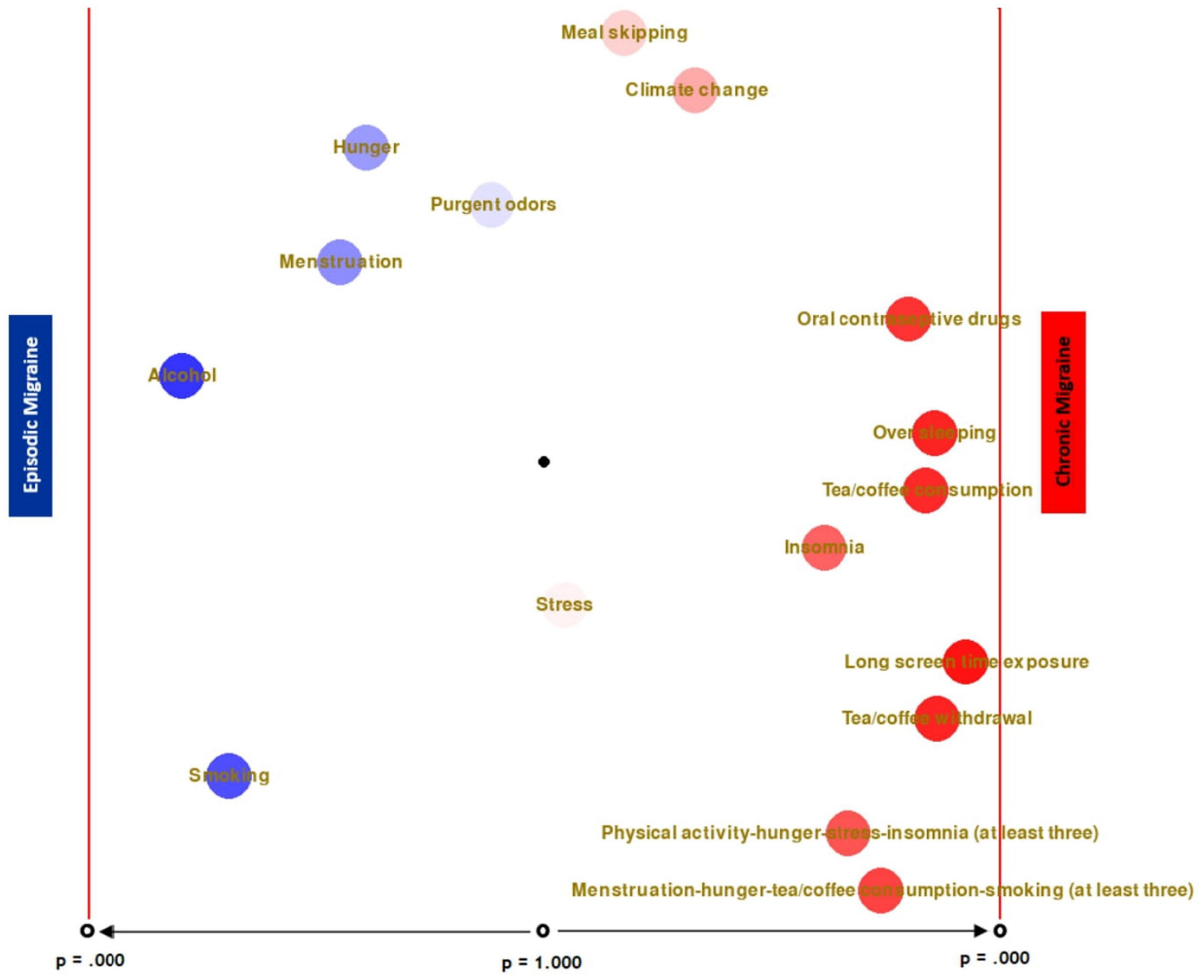
Comparison of triggers between the migraine groups and treatment response.

As a result of a series of chi-squared analysis, there is no significant difference in the rates of benefit from treatment depending on the presence of triggers in both episodic and chronic migraine groups (all *p*s > 0.05). However, with the presence of a greater number of triggers in the chronic migraine group, there is an increase in significance in favor of no treatment benefit (see Figure 2).

### Analysis of environmental factors/triggers that tend to coexist according to the migraine groups

One of the most significant differences between migraine groups is that in the chronic migraine group, travel and physical activity are in the second cluster, while in the episodic migraine group, physical activity is in the second cluster and travel is in the first cluster.

**Figure 3:**
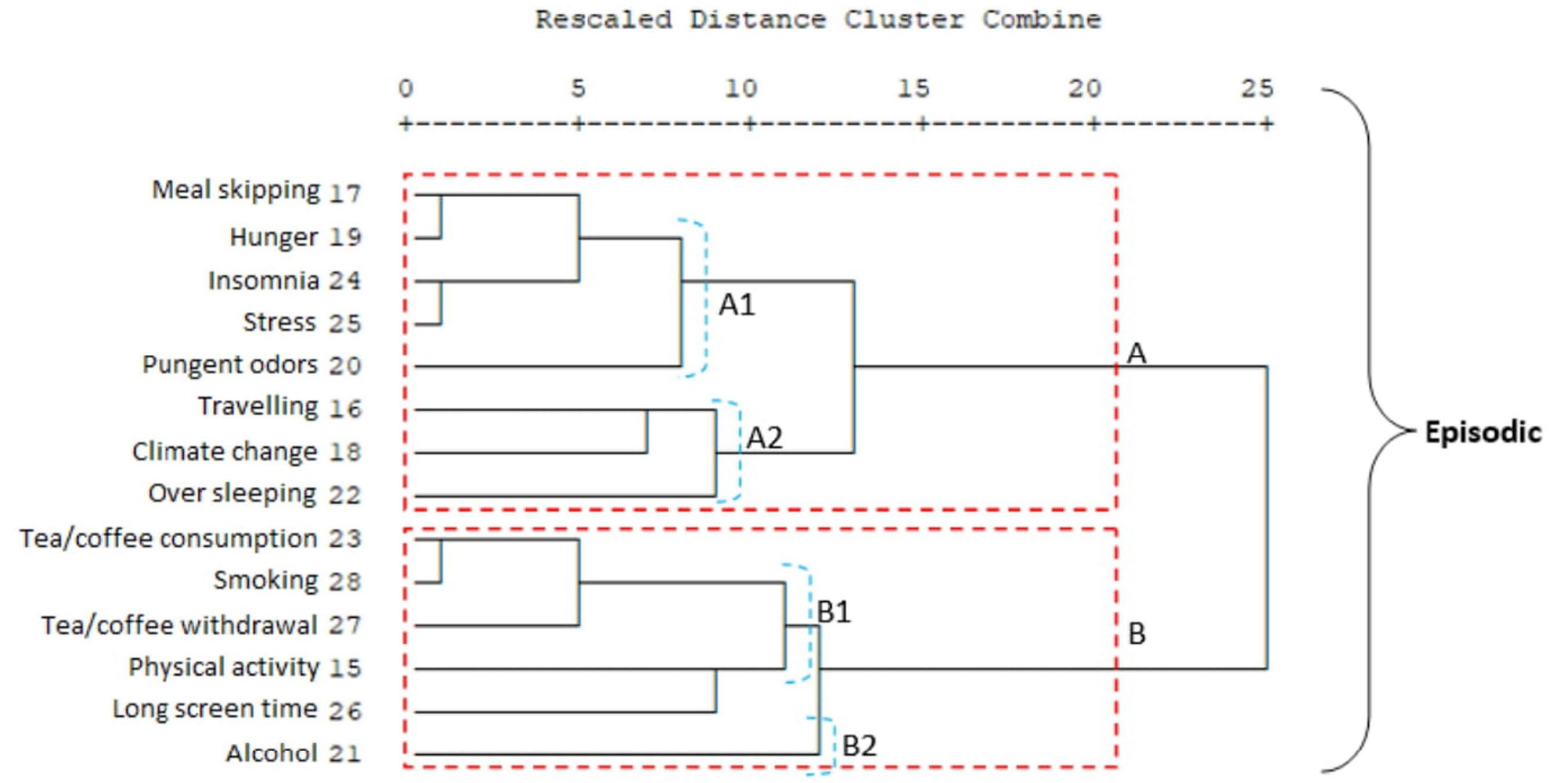
Cluster analysis of the episodic migraine group in terms of triggers.

**Figure 4:**
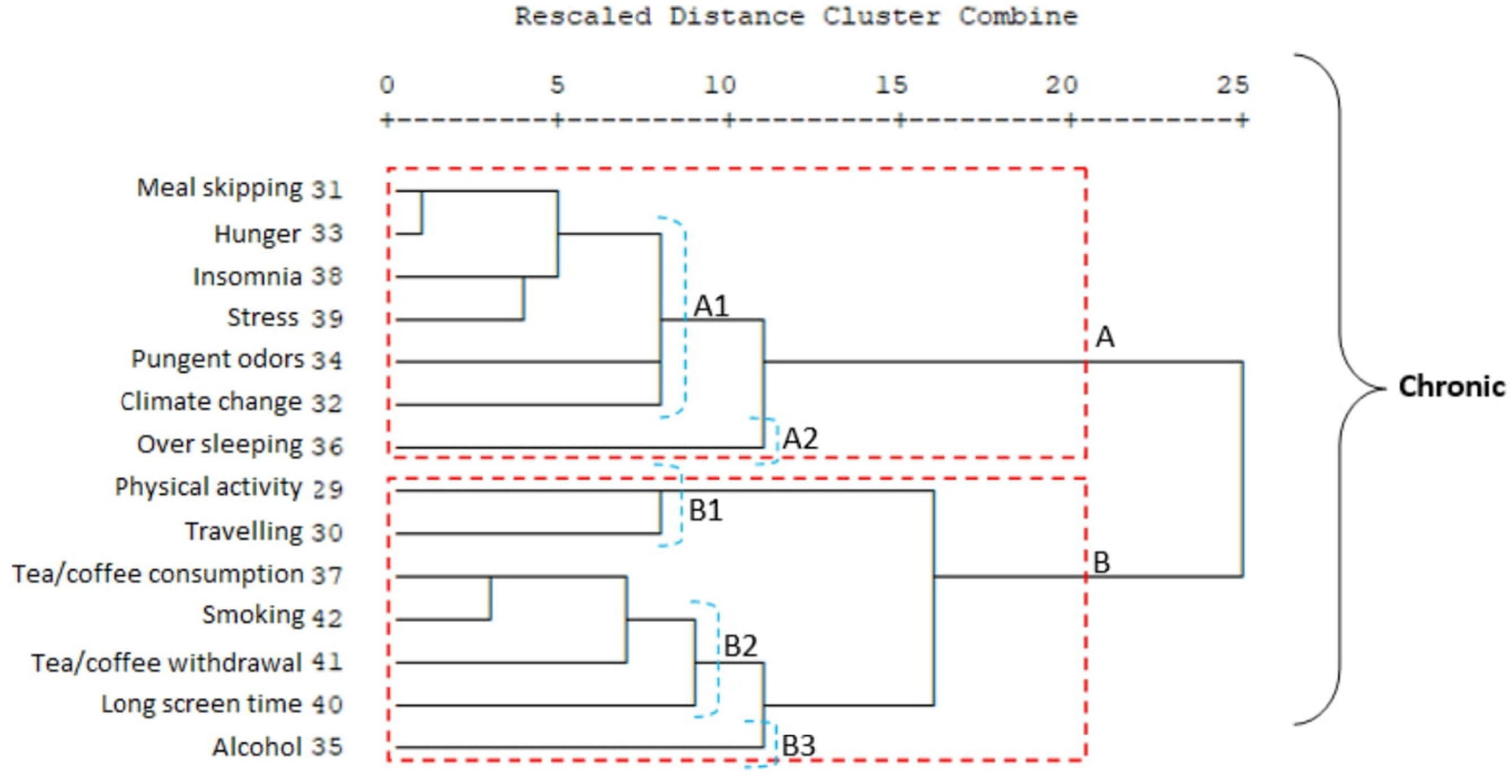
Cluster analysis of the chronic migraine group in terms of triggers.

**Figure 5:**
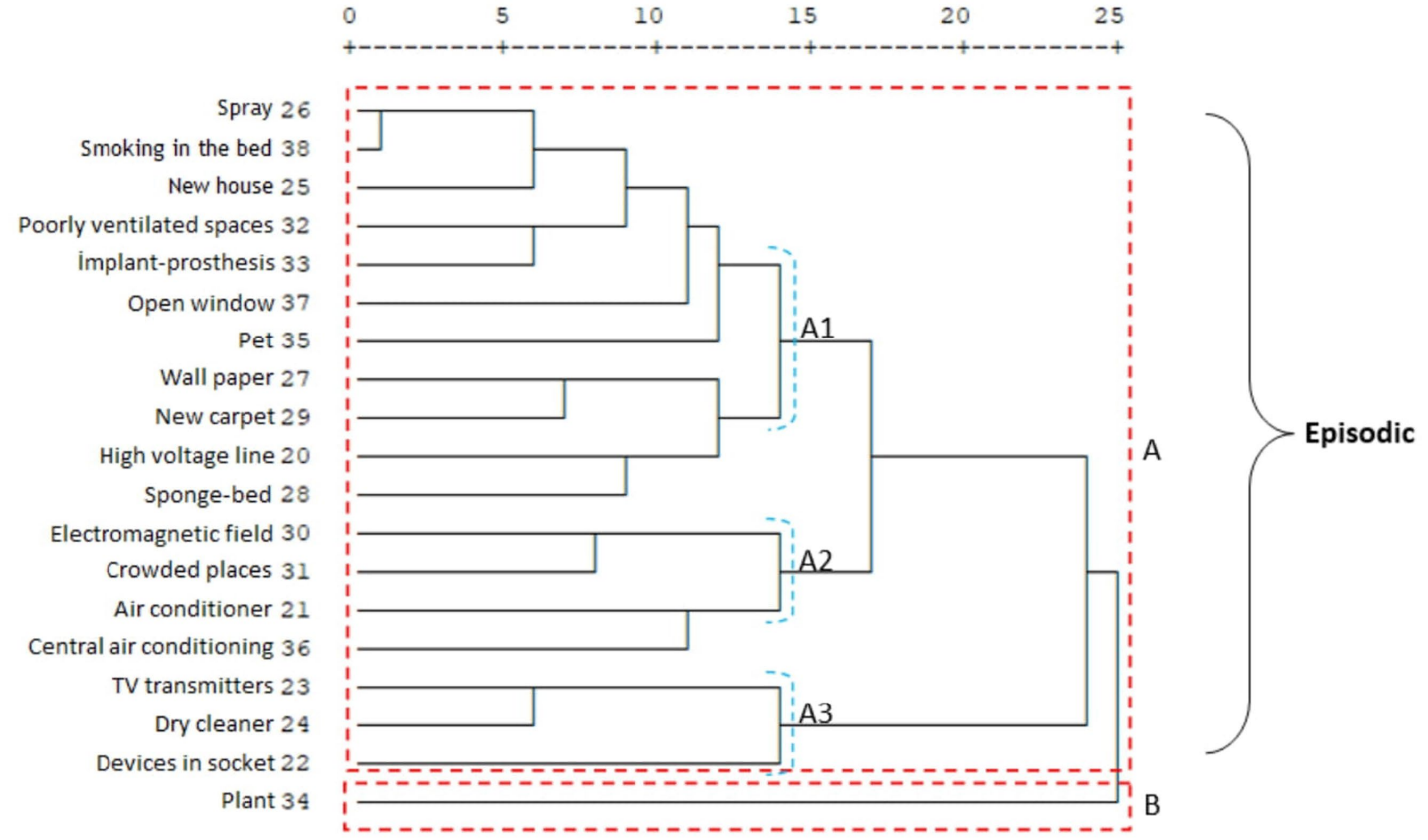
Cluster analysis of the episodic migraine group in terms of environmental factors.

**Figure 6:**
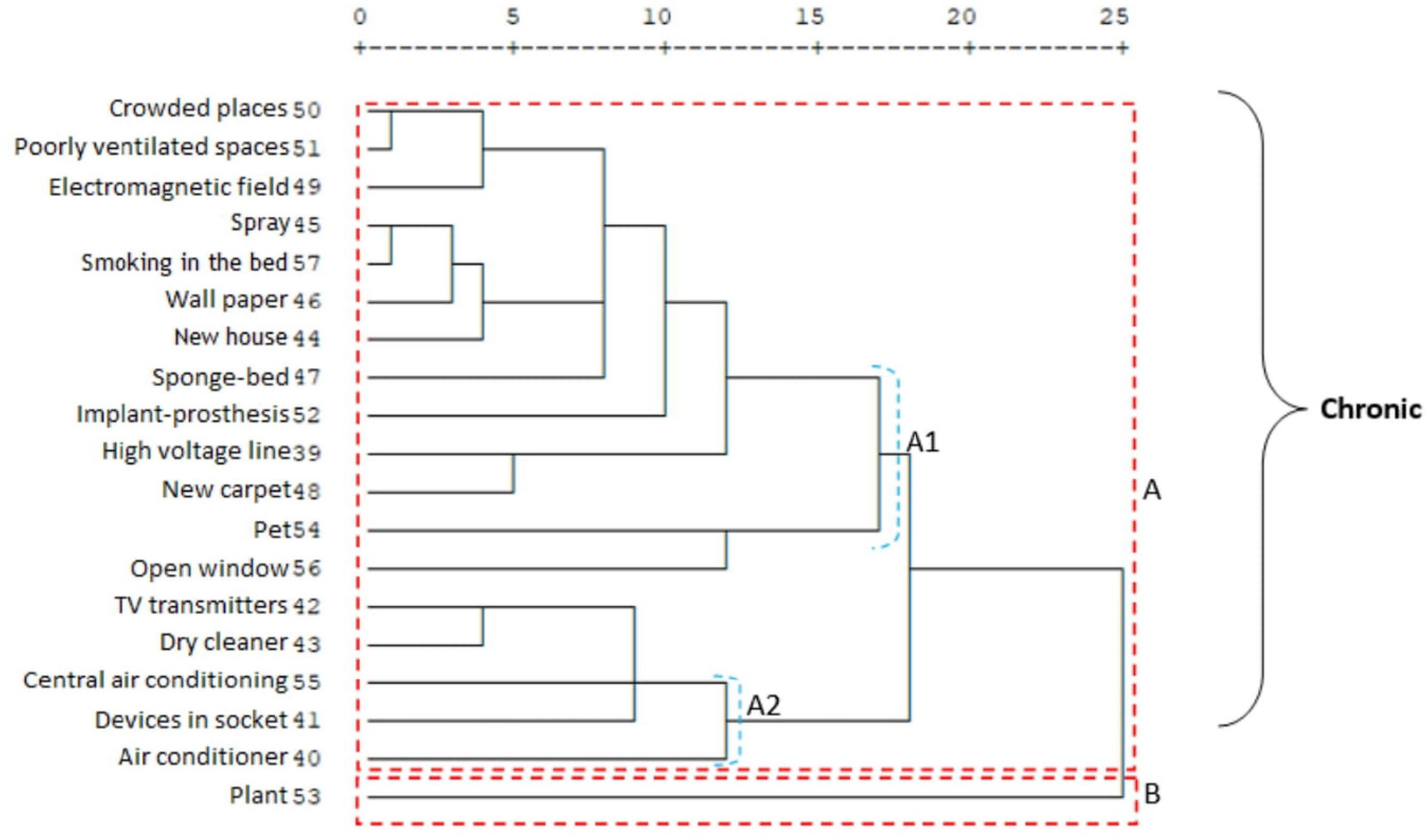
Cluster analysis of the chronic migraine group in terms of environmental factors.

An important difference between the two migraine groups in terms of the subsets of clusters A and B is that in the chronic migraine group, the travel and physical activity items form a separate group within cluster B (B1). In addition, in the chronic migraine group, cluster A, which is the item of sleeping too much, forms a separate group on its own (A2); in the episodic migraine group, sleeping too much, traveling, and weather changes constitute the A2 cluster (Figures 3, 4). It was observed that environmental factors were collected in two main clusters, A and B, in both migraine groups. In both groups, plants form a separate cluster (B), while other environmental factors form a separate cluster (A). In the episodic migraine group, cluster A includes three subclusters (A1, A2, and A3). Subcluster A in the chronic migraine group includes two subclusters (A1 and A2) (Figures 5, 6).

### Comparison of migraine groups according to medication overuse

It was found that the participants in the chronic migraine group used more painkillers than the participants in the episodic migraine group (*p* < 0.001). In addition, it was found that the participants who stated that physical activity, oversleeping, tea/coffee consumption, and smoking were among the triggers had a higher monthly amount of medication use than the participants who did not specify (*p* = 0.039, *p* < 0.001, *p* = 0.002, *p* = 0.001) (Table 5).

**Table 5:** Relationship between triggers and medication overuse.

	*X̅* (*SD*)	*t*	*p*
Physical activity			
Yes	17.64 (17.03)	2.079	0.039
No	14.13 (14.27)		
Oversleeping			
Yes	18.26 (17.10)	6.48	0.000
No	11.78 (12.09)		
Tea/coffee consumption			
Yes	20.85 (19.21)	3.08	0.002
No	14.42 (14.44)		
Smoking			
Yes	21.39 (18.77)	3.32	0.001
No	14.26 (14.43)		

## Discussion

Migraine is a prevalent and debilitating neurological condition that can significantly impact an individual’s quality of life. While various genetic and environmental factors contribute to the development and chronification of migraine, the role of specific environmental factors in the chronification of this condition remains inadequately explored. This article aims to address this research gap by focusing on the impact of manageable and unmanageable factors, such as inadequate living environments, exposure to allergens, electromagnetic fields, and stress factors, on the chronicity of migraine.

### Migraine features/phenotypes and environmental factors/triggers

Triggering factors for migraine are defined as endogenous or exogenous elements that are associated with an increased likelihood of attacks occurring within a short period of time. Triggers, which are identified in more than half of patients, must be distinguished from prodromal symptoms that precede the headache phase (10). Good identification of triggers can lead to effective avoidance behavior and contribute to treatment.

In a study conducted by Zebenholzer et al. (11), pain diaries kept by 327 individuals with migraine were evaluated. In this study, when lifestyle and triggers were evaluated separately, it was shown that 41 of 45 lifestyle factors (91%) were statistically significantly related. In this study, excessive sleep and physical activity were not considered triggers for headache. However, in our study, the chronic migraine group identified excessive sleep as a trigger more than the episodic group. This may suggest that excessive sleep is a risk factor for episodic migraine becoming chronic. Smoking, which is also one of the modifiable triggers, was also defined as a significant trigger in our study, as shown previously (11, 12). Additionally, unlike our study, when the relationship between the frequency of MHD and MMD and trigger/environmental factors was investigated, menstruation, which is an unchangeable risk factor shown in many studies, was also identified as a significant trigger for the frequency of MMD in our study (13). According to our data, exposure to high-voltage lines and crowded places was defined as a trigger for episodic migraine. Being in a crowded place was identified as a trigger in a recent study (14). However, in this study, no differentiation was mentioned for the CM or EM groups. Additionally, proximity to high-voltage areas has not been studied in detail before. Assessment of individuals who identify these modifiable risk factors may facilitate treatment management.

When the triggers that may have an impact on the frequency of MHD are evaluated, unlike MMD, TV transmitters, physical activity, stress, and lack of tea and coffee were evaluated as significant triggers. In our study, unlike other studies, the effect of triggers not only on migraine pain but also on general headaches was studied. The most important contribution to this situation may be the benefit that a detailed evaluation of both headache types will bring to pain management. As a result, in this study, while only menstruation and smoking were the triggers for MMD frequency, no significance was observed in terms of the associations between all factors. However, while TV broadcasting, physical activity, stress, and tea–coffee staleness were separately defined as triggers for MHD, their coexistence was also observed to be significant for the frequency of MHD.

The presence of at least three of physical activity, hunger, stress, insomnia, or the combination of physical activity and travel is a significant trigger group for MHD. The tendency for these triggers to act together has been considered highly valuable. It is thought that the contribution of questioning and preventing other well-defined triggers, which are likely to coexist in the presence of a particular trigger, to migraine treatment management cannot be denied. Physical activity has previously been shown to be a trigger, and the demonstration that it can be found together with other triggers adds a distinctive feature to this study (15).

The study observed significant differences in migraine phenotype between episodic and chronic migraine groups, including attack frequency, severity, and certain triggers.

Triggers such as stress, physical activity, and caffeine withdrawal were associated with increased migraine frequency and severity, particularly in chronic migraine patients.

Hierarchical cluster analysis revealed distinct patterns of trigger coexistence in episodic and chronic migraine groups, providing insights into potential treatment strategies tailored to individual trigger profiles.

### Environmental factors/triggers and response to treatment

It is thought that this study, in which the relationship between response to treatment and environmental factors/triggers is comprehensively evaluated, provides important data for big data from these different perspectives. It has been shown that patients in the CM group who cited new homes as a trigger had less response to treatment. In the EM group, those who cited TV transmitters, dry cleaning, live plants, plug-in devices, alcohol, and cigarettes as triggers were defined as more resistant to treatment. The differentiation of these risk factors in both migraine groups is considered quite remarkable and very valuable. When these data are evaluated together with the fact that both episodic and chronic migraines turn into each other over time, it should be kept in mind that the triggers should be re-examined in both groups when there is no response to treatment.

### Different perspective on medication overuse in migraine

Alcohol is one of the most frequently cited migraine triggers. However, it has previously been shown that people with migraine are less likely to drink, and alcohol is not linked to migraine (16). However, it is also known that people with migraine may develop headaches with alcohol or red wine (17). The relationship between alcohol and migraine has a complex structure (18), and in our study, less response to treatment was found in the episodic migraine group that cited alcohol use as a trigger.

When cluster analyses of trigger/environmental factors in episodic and chronic migraine groups are examined, again, alcohol use is a separate subset of both groups. When this situation is evaluated together with other data, alcohol should be evaluated separately for the chronicity of migraine and response to treatment. In addition, the clustering of triggers that can be associated with addiction, such as smoking, alcohol, tea, and coffee consumption, and long screen time, is very important data for both episodic and chronic migraine. The relationship between internet addiction, long screen time, and migraine has been previously shown (19). Additionally, chronic migraine is often associated with medication-overuse headaches. The endocannabinoid system is involved in modulating pain, including headache, and is involved in the common neurobiological mechanism underlying drug addiction and the reward system. Therefore, there seems to be an undeniable relationship between chronic headache and/or an adaptive behavior caused by excessive medication use (20). In addition to the association between drug overuse, which is a modifiable trigger, and migraine, a relationship between drug overuse and addiction has been previously demonstrated (21). In our study, medication overuse was observed to be more common in chronic migraine patients, while medication overuse headaches were more common in groups reporting excessive sleeping, smoking, and tea and coffee consumption as triggers. Although this study did not focus on the relationship between drug overuse and migraine, it is thought that showing these secondary results comprehensively, that is, the relationship between addictive triggers in migraine, is very important in terms of emphasizing the importance of an individualized approach in the evaluation of patients.

## Conclusion

The study’s use of comprehensive statistical methods, including hierarchical cluster analysis and chi-squared analysis, provides novel insights into the complex interplay between environmental factors, migraine phenotype, and treatment response. This methodological approach provides a more nuanced understanding of migraine pathophysiology and treatment strategies. The study’s findings underscore the importance of incorporating environmental factors into migraine management strategies. Clinicians should consider assessing and addressing environmental triggers as part of routine clinical practice to optimize treatment efficacy and improve patient outcomes.

## Data Availability

The raw data supporting the conclusions of this article will be made available by the authors, without undue reservation.
